# Proteomic Applications in the Study of Human Mesenchymal Stem Cells

**DOI:** 10.3390/proteomes2010053

**Published:** 2014-02-07

**Authors:** Jesús Mateos, Pablo Fernández Pernas, Juan Fafián Labora, Francisco Blanco, María del Carmen Arufe

**Affiliations:** 1 Rheumatology Division, ProteoRed/ISCIII, INIBIC-Hospital Universitario A Coruña, A Coruña 15006, Spain; E-Mail: jesus.mateos.martin@sergas.es (J.M.); fblagar@sergas.es (F.B.); 2 CIBER-BBN, INIBIC-Hospital Universitario A Coruña, A Coruña 15006, Spain; E-Mail: pablofpernas@gmail.com (P.F.P.); juanlaru_15@hotmail.com (J.F.L.); 3 Department of Medicine, University of A Coruña, A Coruña 15006, Spain; 4 Department of Medicine, University of Santiago de Compostela, Santiago de Compostela 15782, Spain

**Keywords:** mesenchymal stem cell, proteomic analysis, characterization, differentiation

## Abstract

Mesenchymal stem cells (MSCs) are undifferentiated cells with an unlimited capacity for self-renewal and able to differentiate towards specific lineages under appropriate conditions. MSCs are, *a priori*, a good target for cell therapy and clinical trials as an alternative to embryonic stem cells, avoiding ethical problems and the chance for malignant transformation in the host. However, regarding MSCs, several biological implications must be solved before their application in cell therapy, such as safe *ex vivo* expansion and manipulation to obtain an extensive cell quantity amplification number for use in the host without risk accumulation of genetic and epigenetic abnormalities. Cell surface markers for direct characterization of MSCs remain unknown, and the precise molecular mechanisms whereby growth factors stimulate their differentiation are still missing. In the last decade, quantitative proteomics has emerged as a promising set of techniques to address these questions, the answers to which will determine whether MSCs retain their potential for use in cell therapy. Proteomics provides tools to globally analyze cellular activity at the protein level. This proteomic profiling allows the elucidation of connections between broad cellular pathways and molecules that were previously impossible to determine using only traditional biochemical analysis. However; thus far, the results obtained must be orthogonally validated with other approaches. This review will focus on how these techniques have been applied in the evaluation of MSCs for their future applications in safe therapies.

## 1. Introduction

Mesenchymal stem cells (MSCs) are multipotent cells with an important potential in human regenerative medicine because of their ability to migrate to sites of injury [1], their capability of suppressing the immune response [2] and their accessibility in large numbers from the patient’s own bone marrow or fat tissue. It has been increasingly observed that the transplanted MSCs do not necessarily engraft and differentiate at the site of injury, but might exert their therapeutic effects through secreted trophic signals [3]. MSCs secrete a variety of autocrine/paracrine factors that make up the secretome, which supports regenerative processes in the damaged tissue, induces angiogenesis, protects cells from apoptotic cell death and modulates the immune system. The MSC secretome has become a subject of intensive proteomic profiling in the search for and identification of released factors and microvesicles that might be applicable in regenerative medicine. Jointly with the methods for MSC isolation, expansion and differentiation, proteomic secretome analysis of MSC has been increased in use, mainly due to the extensive development of protein separation techniques and mass spectrometry, recently reviewed by Skalnikova *et al*. [4]. This review will focus on the study of the intracellular proteome.

The term proteomics encompasses all research methodologies aimed at qualitative and quantitative study of the proteins, or proteome, present in a cell type, tissue or organism at a given stage of development [5]. In the last decade, there has been an exponential increase in the use of these techniques in translational research, due in large part to the progress in state-of-the-art mass spectrometry. With this technique, developed in the middle of the last century [6], it is possible to calculate, with remarkable accuracy, the mass/charge ratio of any compound that can ionize, as well as the mass/charge ratio of the fragments originated by the collision of the compound with an inert gas. The accuracy in the measurement is so high that it is actually possible to identify the compound. In the case of proteomics, mass spectrometers are designed to identify and quantify peptides and proteins in a very sensitive and high-throughput manner. It has been widely accepted that proteomics can never replace, but complements, genomic information [7,8]. First, unlike what happens in genomics, proteomics studies lack a technique, such as polymerase chain reaction (PCR), to amplify these very low abundance molecules for study. This greatly complicates the study of very scarce proteins, such as growth factors or cytokines [9]. Unfortunately, the wide dynamic range of protein concentration causes the most abundant signal to mask the signal of low-abundance molecules. Finally, post-translational modifications, such as phosphorylation, glycosylation, *etc*., can completely change the function of a protein, but the total amount thereof does not vary. Due, at least in part, to these limitations, it is now required that the data obtained in individual samples be tested using proteomic techniques, including Western blot or enzyme-linked immuno sorbent assay (ELISA) or even genomic techniques, such as real-time PCR (RT-PCR).

The protein expression profile of MSCs may reveal potential hazards associated with senescence and tumoral transformation that may occur during culture. Proteomic is a valuable tool for human MSC characterization following physiological modifications of the phenotypes of MSCs and identification of possible changes occurring during expansion. Mass spectrometry-based comparative membrane proteomics can enable the identification of novel cancer biomarkers by distinguishing proteins that change membrane localization between normal and malignant tissues and cells. The combination of analyzers and other types of available components has led in recent years to a long list of devices designed specifically for each type of molecule. Specifically, the range of platforms designed for the analysis of peptides and proteins has been adapted specifically to different qualitative and quantitative techniques (Table 1). This review describes proteomic techniques currently applied or prospectively applicable to MSC studies.

**Table 1:** Studies of mesenchymal stem cells (MSCs) using quantitative proteomic techniques.

Proteomic Technique	MSC Source	Biological significance	Instrument	Ref.
2D-LC-MS/MS	Bone marrow	Characterization	Q-TOF	[10]
2DE-MALDI-TOF/TOF MS	Umbilical cord blood	Characterization	MALDI-TOF/TOF	[11]
2DE-MALDI-TOF-MS	Amniotic fluid	Characterization	MALDI-TOF	[12]
2D-LC-MALDI-MS	Bone marrow	Characterization	MALDI-TOF	[13]
2DE and combined MS and MS/MS	Bone marrow	Characterization	MALDI-TOF/TOF	[14]
2DE and combined MS and MS/MS	Umbilical cord	Characterization	MALDI-TOF/TOF	[14]
2DE and combined MS and MS/MS	Placenta	Characterization	MALDI-TOF/TOF	[14]
DIGE-MALDI-TOF/TOF	Bone marrow	Characterization	MALDI-TOF/TOF	[15]
2DE-PMF	Bone marrow	Characterization	MALDI-TOF/TOF	[16]
2DE-PMF	Bone marrow	Characterization	MALDI-TOF/TOF	[17]
GELFREE-LC-MALDI-TOF/TOF	Bone marrow	Characterization	MALDI-TOF/TOF	[18]
2DE-MALDI-TOF-MS	Bone marrow	Extension culture	MALDI-TOF	[19]
2DE-MALDI-TOF-MS	Bone marrow	Extension culture	MALDI-TOF	[20]
2DE-MALDI-TOF-MS/MS	Bone marrow	Extension culture	MALDI-TOF	[21]
2DE-MALDI-TOF-MS/MS	Bone marrow	Extension culture	MALDI-TOF	[22]
SELDI-TOF-MS	Adipose tissue	Extension culture	SELDI-TOF	[23]
2DE coupled MS	Bone marrow	Extension culture	Q-TOF	[24]
2DE-MALDI-MS	Bone marrow	Senescence	MALDI-TOF	[25]
2DE-ESI-Q-TOF-MS/MS	Bone marrow	Senescence	Q-TOF	[26]
DIGE-MALDI-TOF-MS	Bone marrow	Extension culture	MALDI-TOF	[27]
2DE-ESI-MS/MS	Bone marrow	Differentiation	Q-TOF	[28]
LC-MS/MS	Bone marrow	Differentiation	Q-TOF	[29]
DIGE-MALDI-TOF-MS	Bone marrow	Differentiation	MALDI-TOF	[30]
2DE-MALDI-TOF-MS	Bone marrow	Differentiation	MALDI-TOF	[31]
2DE-ESI-Q-TOF-MS/MS	Umbilical cord blood	Differentiation	Q-TOF	[32]
DIGE-MALDI-TOF/TOF-MS/MS	Adipose tissue	Differentiation	MALDI-TOF/TOF	[33]
2DE-MALDI-TOF/MS	Umbilical cord blood	Differentiation	MALDI-TOF	[34]
LC-coupled MS/MS	Intervertebral disc	Differentiation	LTQ	[35]
LC-coupled MS/MS	Bone marrow	Differentiation	LTQ-Orbitrap	[36]
DIGE- MALDI-TOF/TOF-MS/MS	Umbilical cord stroma	Differentiation	MALDI-TOF	[37]
SILAC-LC-MALDI-TOF/TOF-MS/MS	Bone marrow	Differentiation	MALDI-TOF/TOF	[38]
DIGE-MALDI-TOF-MS	Bone marrow	Differentiation	MALDI-TOF	[39]
SILAC-LC-MS/MS	Bone marrow	Differentiation	LTQ-Orbitrap	[40]
DIGE-IEF-MALDI-MS/MS	Bone marrow	Cell Therapy	MALDI-TOF/TOF	[41]
2DE-MALDI-TOF-MS	Bone marrow	Cell Therapy	MALDI-TOF	[42]
DIGE-MALDI-TOF/TOF-MS/MS	Bone marrow	Cell Therapy	MALDI-TOF/TOF	[43]
SDS-PAGE-LC coupled MS/MS	Bone marrow	Cell Therapy	LTQ	[44]
LC-coupled MS/MS	Bone marrow	Cell Therapy	LTQ-Orbitrap	[36]
DIGE-MALDI-TOF-MS	Bone marrow	Cell Therapy	MALDI-TOF	[45]

2DE, two-dimensional electrophoresis; MALDI, matrix-assisted laser desorption ionization; TOF, time of flight; PMF, peptide mass fingerprinting; DIGE, difference in-gel electrophoresis; SELDI, surface enhanced laser desorption/ionization; ESI, electrospray ionization; SILAC, stable isotope labeling by/with amino acids in cell culture; IEF, iso-electric focusing; SDS-PAGE, sodium dodecyl sulfate polyacrylamide gel electrophoresis; LTQ, linear trap quadrupole.

## 2. Proteomic Techniques

Proteins extracts coming from cultured cells are highly complex protein samples that present a wide dynamic range of concentrations [46]. Fractionation of the sample is therefore necessary to reduce its complexity. Protein quantification performed by colorimetric or fluorometric assays is one of the weaknesses of the entire proteome flow, because there is currently no universal method to quantify any sample with high accuracy and reproducibility. After fractionation by electrophoretic techniques, isoelectric focusing or chromatographic analysis, subsequent identification and quantification of the peptides allows the identification and quantification of the original proteins using certain algorithms. In some cases, directed digestion is initially performed in solution, followed by peptide fractionation as liquid chromatography (LC), strong cation exchange (SCX) or the off-gel separation of peptides. Alternatively, fractionation of proteins by gel size or isoelectric point is performed first as sodium dodecyl sulfate polyacrylamide gel electrophoresis (SDS-PAGE), two-dimensional polyacrylamide gel electrophoresis (2D-PAGE) or isoelectric focusing (IEF) followed by digestion. Sometimes, the digestion is performed between two successive fractionations, depending on the sample type and complexity. In any case, fractionation of the studied sample clearly decreases the complexity of the resulting fractions, but also lengthens the total time for analysis.

It is currently accepted that a research project based on proteomics should have two phases. In the initial phase of shaping or profiling, the proteome or protein profile of a particular type of sample, such as a cell line or tissue samples at a given stage of development of MSCs, is determined. One way to do this is by two-dimensional electrophoresis (2DE) with subsequent identification of proteins by peptide mass fingerprinting using a matrix-assisted laser desorption ionization-time of flight (MALDI-TOF) platform. In this technique, developed in the early nineteen-nineties by Henzel *et al*. [47], proteins are separated on acrylamide gels in two dimensions, by isoelectric point and by size and can be identified by the pattern of tryptic peptides, *i.e*., from in-gel digestion with trypsin, which specifically digests protein in lysine and arginine residues. The combination of peptides with a specific ion *m/z* ratio is unique to each protein and depends only on their amino acid sequence and post-translational modifications of these; so, this is called protein finger printing. The study may simply be qualitative, *i.e*., determine which peptides and, therefore, proteins are or are not present in the sample, or quantitative, to determine their relative abundance between the conditions under study. Many diseases are not due to the presence or absence of a specific protein or group of proteins, but to changes in the abundance; in this case, it is necessary, as in most cases, to perform a differential metabolic or chemical labeling of the samples. Once differentially labeled, samples are mixed, and thereafter, it becomes a single process in order to reduce experimental variability and bias, assuming, of course, that a precise quantification of the total amount of protein in the different samples has been done previously.

In a second discovery phase, techniques, such as the difference in-gel electrophoresis (DIGE) [48], a variant of the two-dimensional electrophoresis in which the proteins are labeled with a fluorophore, the isobaric tags for relative and absolute quantification (iTRAQ) or stable isotope labeling by/with amino acids in cell culture (SILAC) are used for relative quantification. The analysis is performed on a selected number of samples (in many cases working with sets or pools of samples) and generates a panel of several tens of proteins or peptides modulated between the different conditions under study and, therefore, to be candidates for potential targets, such as a possible drug treatment. Among the most popular platforms used in this phase, we find the linear trap quadrupole (LTQ)-orbitrap and the MALDI-TOF/TOF or LC-MALDI-TOF/TOF when combined with an off-line chromatographic system. At a later stage of verification, the linear trap quadrupole coupled to Fourier transform ion cyclotron resonance (LTQ-FT) or triple quadrupole-ion trap (QqQ-trap) platforms are used, allowing direct absolute quantification of a specific candidate in a large number of individual samples, which is known as multiple or selected reaction monitoring or assays (MRM and SRM, respectively). This involves the selection of representative peptides of the candidate that must meet certain characteristics and also the synthesis of isotope-labeled versions of that peptides that are spiked in the sample in a known amount, enabling absolute quantification of the protein [49].

In any case, the raw data generated in the analysis of any of the phases are reflected in the mass spectra and fragmentation of peptides, which is a dimensional graphical representation of the different intensities of detected ions against their *m/z* ratio. These data are processed and interpreted by search engines, equipped with powerful software, using complicated algorithms that integrate the raw data with existing protein databases at different sequence repositories (Uniprot, Nextprot, NCBI), allowing the identification and, if appropriate, quantification of the proteins present in the samples analyzed.

**Figure 1:**
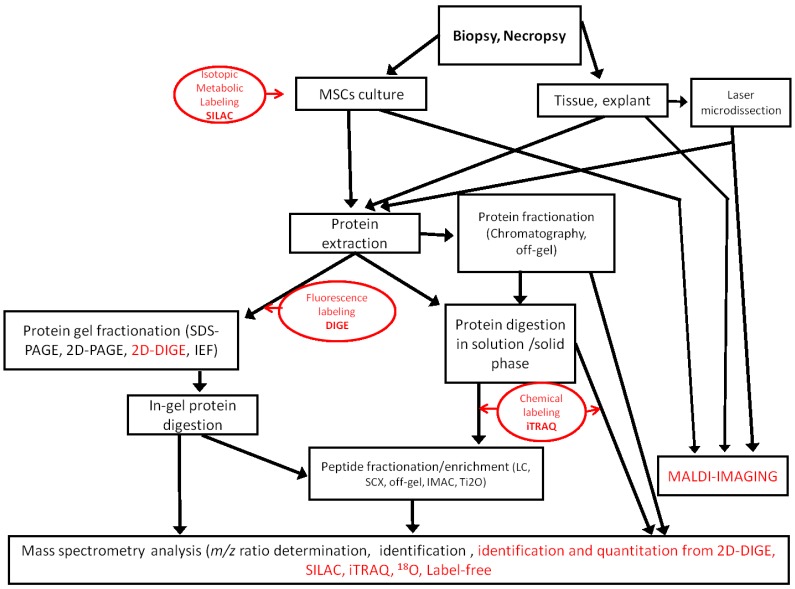
Workflows used in MSC proteomic analysis. Quantitative methods are indicated in red. SCX, strong cation exchange; IMAC, immobilized metal ion affinity chromatography; iTRAQ, isobaric tags for relative and absolute quantification.

The proteomic approach generates informative data on the expression and post-translational modifications of proteins that are useful to assess the true potential of MSCs in regenerative medicine. No matter which technologies are used, proteomic analysis is always a challenge, because the proteome is extremely diverse [50], changes with time and is highly sensitive to pre-analytical conditions (Figure 1).

## 3. Characterization of Mesenchymal Stem Cells

To date, 2DE gel analysis has been the most used proteomic approach for determining cell surface markers of MSCs [51]. The goal is to compare cells from different origins, to follow their differentiation and to ultimately define a specific MSC proteomic signature. One important initial task is the optimization of 2DE protocols, so that they are robust enough to be used in a multisite project. Provansal *et al*. detailed several thorough protocols that can be used for MSCs in culture [50].

Salasznyk *et al*. [10] identified markers for two cell populations, analyzed the expression of human MSC proteins and compared them to those of human osteoblasts using 2D-LC-MS/MS. Among the 755 different proteins identified in both cell populations, two sets of proteins, 247 found only in human MSCs and 158 in human osteoblast cells, were identified. Substantial differences in clusters of proteins responsible for calcium-based signaling and cell adhesion were found between the two cell types.

Feldmann *et al*. [11] achieved protein identification using MALDI-TOF-MS and gel-matching with previously identified databases in their characterization of MSCs from umbilical cord blood after 2DE. Roughly 205 molecules were identified representing 145 different proteins and 60 isoforms or post-translational modifications. The identified proteins could be grouped into several functional categories, including metabolism, folding, cytoskeleton, transcription, signal transduction, protein degradation, detoxification, vesicle/protein transport, cell cycle regulation, apoptosis and calcium homeostasis.

Roubelakis *et al*. [12], using 2DE and the MALDI-TOF-MS approach, have generated, for the first time, a protein map of cultured amniotic fluid MSCs by identifying 261 proteins. They directly compared the amniotic fluid MSC protein map with that of cultured MSCs from bone marrow and found that the functional pattern of the identified proteins from both sources was similar. However, cultured MSCs from amniotic fluid displayed a number of unique proteins related to proliferation and a primitive phenotype, which may be attributable to the distinct features of the two MSC types.

Protein profiling of MSC clonal populations was conducted by 2D-LC-MALDI-MS by Mareddy *et al*. [13]. A total of 83 proteins was identified with high confidence, of which 11 showed differential expressions between subpopulations, including cytoskeletal and structural proteins, calcium binding proteins, cytokinetic proteins and members of the intermediate filament family. This study generated a proteome reference map of bone marrow MSCs from the clonal populations, which will be valuable to better understand the underlying mechanism of MSC self-renewal and differentiation.

Li *et al*. [14], using 2DE and combined MS and MS/MS analysis, identified six differentially expressed proteins among MSC samples derived from bone marrow, umbilical cord and placenta, with five of them known to be involved in cell migration as either migration enhancing or inhibiting proteins. Consistent with their migration capacity, the levels of migration enhancing proteins, including cathepsin B, cathepsin D and prohibitin, were significantly lower in MSCs from umbilical cord when compared with those MSCs from bone marrow and MSCs from placenta. A higher expression for migration inhibiting proteins, including plasminogen activator inhibitor-1 (PAI-1) and manganese superoxide dismutase, was found in MSCs from umbilical cord. They also showed that the overexpression of PAI-1 impaired the migration capacity for bone marrow and placenta MSCs, while silencing of PAI-1 enhanced the migration capacity of umbilical cord MSCs. Their study indicated that migration-related proteins are pivotal in the chain of events governing the migration capacity of MSCs.

Jaishankar *et al*. [15] reported a nuclear proteomic analysis of human embryonic and bone marrow-derived MSCs. Their proteomic screen highlighted a five-fold difference in the expression of Reptin52. They showed, using 2D-DIGE, that Reptin52 is more abundantly expressed in human embryonic stem cells than human MSCs. Moreover, they observed differential expression of Pontin52 and beta-catenin-proteins known to interact with Reptin52, known regulators of beta-catenin, further supporting a role for Wnt signaling in stem cell self-renewal and proliferation.

The expression of a specific set of cell surface cluster differentiation (CD) markers, (CD13, CD29, CD44, CD73, CD90, CD105 and CD166) and the absence of hematopoietic stem cell markers (CD34, CD45, CD117 [52], HLA class I and HLA-DR antigens [16,17]) strongly support the characterization of MSCs using 2DE-PMF. In this regard, it is worth noting that different groups reach similar conclusions using different proteomic platforms.

Mindaye *et al*. [18] achieved the proteomic analysis of membrane proteins, which is challenging and notably underrepresented in proteomic studies, due to the difficulty in the extraction and isolation of lipophilic proteins embedded in lipidic layers. They introduced a new approach, including high pressure-assisted membrane protein extraction, protein fractionation by gel-eluted liquid fraction entrapment electrophoresis [18] and the combined use of liquid chromatography MALDI and ESI tandem mass spectrometry. This report presented the first comprehensive proteomic analysis of the membrane proteome of undifferentiated and culture-expanded human MSCs from bone marrow obtained from different human donors. This new workflow approach enabled them to identify at least two-fold more membrane proteins compared to previous published works. A total of 84 cell surface CDs were identified, including 14 newly-identified CDs.

From these works, we can conclude that cellular compartment pre-fractionation drives to a better characterization of the stem cell populations, which may be defined in the near future by their proteomic profile regardless of their origin, age or stage.

## 4. *Ex Vivo* Cultivation of Mesenchymal Stem Cells

MSCs hold great promise for cell-based therapeutic use, because of their multipotency and the existence of simple methods for *in vitro* expansion. However, during *in vitro* expansion, MSCs will age and lose their multipotency and proliferation capability. Several studies have provided evidence that homogeneous MSCs preparations can be reproducibly isolated under standardized conditions; however, culture conditions exert a major impact on the transcriptome, proteome and cellular organization of MSCs. Sun *et al*. [19] used 2DE-MALDI-TOF-MS to perform an analysis during the serial subculture of human MSCs. The expression of 12 polypeptides was consistently differentially regulated (eight upregulated and four downregulated) during serial subculture until the seventh passage. The profile changes were concentrated on proteins related to cell cycle, cell morphology and cell proliferation. The data indicated that MSCs underwent morphological changes and a decline in proliferation over the course of serial cultivation. Of the differentially regulated proteins, cytoskeletal components, including annexin A1 and A2, were upregulated, whereas metabolic, synthetic and degradation pathway-related proteins, such as T-complex protein 1 alpha and T-complex protein 1 gamma, were downregulated during the serial subculture of the isolated human MSCs. Wagner *et al*. [20] using 2DE, also identified 136 protein spots from MALDI-TOF-MS corresponding to the differential protein expression of two human bone marrow populations cultured in two different conditions. Proteins involved in metabolism were more highly expressed in low glucose media, whereas proteins involved in development, morphogenesis, extracellular matrix and differentiation were more highly expressed in a commercial medium.

Lazzarotto-Silva *et al*. [21] compared and analyzed MSCs from human bone marrow at different culture passages using 2DE-MALDI-TOF-MS/MS and observed similar results in all cultures at all passages, suggesting a high degree of similarity among them. The same result was found by Binato *et al*. [22] after image analysis demonstrated that MSCs would have similar protein expression patterns at the first passage. These results suggested that the protein profile of human MSC cultures derived from different passages and different donors were equivalent. However, changes in the proteomic profile of different tissue-derived MSCs during passages in culture have been evaluated using surface enhanced laser desorption/ionization-time of flight-mass spectrometry (SELDI-TOF-MS) by Capra *et al*. [23]. This group evaluated the presence of stable molecular markers in adipose tissue-derived MSCs and found changes in the proteomic phenotype following prolonged *in vitro* culture. The protein with the greatest change in expression during cell culture was identified as calcyclin.

Lee *et al*. [24] used 2DE coupled to MS to identify differentially expressed proteins at the cell membrane level in MSCs growing in basic fibroblast growth factor (bFGF) containing medium; a total of 15 differentially expressed proteins were identified, of which nine of them were upregulated and six downregulated. The expression level of three actin-related proteins, F-actin-capping protein subunit alpha-1, actin-related protein 2/3 complex subunit 2 and myosin regulatory light chain 2, was confirmed by complementary analysis. The results indicated that the expression levels of these there actin-related proteins were important to the bFGF-induced morphological change of MSCs.

Madeira *et al*. [25] studied the molecular mechanisms underlying cellular senescence resulting from extended *ex vivo* cultivation of bone marrow MSCs; they used 2DE-MALDI-MS to demonstrate significant evidence of culture-induced senescence. Proteins involved in cellular structure, the structure of the cytoskeleton, folding and stress response were less abundant in cells with advanced senescence, while proteins involved in energy metabolism, cell cycle regulation, aging and apoptosis were more abundant.

Several studies have reported that caloric restriction increases the proliferation of MSCs and decreases apoptosis. Kim *et al*. [26] examined the effect of low glucose on human bone marrow MSC proliferation compared with that under normal glucose conditions to learn if calorie restrictions modify the proteomic profile of MSCs in culture. 2DE was utilized, and the results found that calorie restriction does not have a significant effect on cell proliferation, reactive oxygen species generation, glucose consumption, population doublings and adipogenic differentiation of MSCs. However, they identified three upregulated proteins and seven downregulated proteins. These results indicate that calorie restriction induced differentially expressed proteins, which may provide further information on the aging and differentiation of stem cells.

A study by Kuboki *et al*. [27] focused on the mechanotransduction of MSCs in response to matrix elasticity. Proteomic profiles of MSCs cultured on tissue culture plastic and soft and stiff matrices were determined using DIGE. The results indicated abundance and organization changes in cytoskeletal proteins, as well as differential regulation of important signaling-related proteins, stress-responsive proteins and also proteins involved in collagen synthesis. The expressions of major cytoskeletal proteins, including actin, tubulin and vimentin, of cells cultured on the gels were remarkably changed. Significant downregulation of α-tubulin and β-actin was observed on gel samples in comparison to the rigid tissue culture plates. The abundance of expression of vimentin appeared to be highest in MSCs cultured on hard gels. These results suggest that the substrate stiffness significantly affects the expression levels of the cytoskeletal proteins of MSCs, with implications for cellular integrity.

The proteomic studies done on cultivated MSCs show the high plasticity of these cells, which are able to regulate the expression of proteins related to the generation of energy, oxidative stress, cell cycle and apoptosis in order to adapt to the factors and conditions of culture. Furthermore, the importance of the support used for the culture should be mentioned, since this affects the expression of, mainly, proteins related to cytoskeleton structure and cell attachment.

## 5. The Mesenchymal Stem Cell Differentiation Process

Proteomic techniques help to study changes in the human MSC signaling transduction network during early differentiation lineage commitment. Several works have demonstrated the value of proteomic tools for studying stem cell differentiation and elucidating the underlying molecular mechanisms.

Wang *et al*. [28] used ESI-MS/MS to identify proteins in 2DE from human bone marrow MSCs cultured with transforming growth factor-β (TGF-β). They generated a proteome reference map of MSCs, and they identified approximately 30 proteins with an increase or decrease in the expression or phosphorylation in response to TGF-β. The proteins regulated by TGF-β included cytoskeletal proteins, matrix synthesis proteins, membrane proteins, metabolic enzymes and others. TGF-β increased the expression of smooth muscle alpha-actin and decreased the expression of gelsolin. Overexpression of gelsolin inhibited TGF-β-induced assembly of smooth muscle alpha-actin. On the other hand, reduction of gelsolin expression enhanced the assembly of alpha-actin and actin filaments without significantly affecting alpha-actin expression. These results suggest that TGF-β coordinates the increase of alpha-actin and the decrease of gelsolin to promote MSC differentiation.

Foster *et al*. [29] used LC coupled MS/MS to characterize changes in the expression of membrane protein markers before and after short-term induction of osteoblast differentiation in a cell model of human MSCs. They identified 463 unique proteins with extremely high confidence, including all known markers of human MSCs, such as CD71, CD105, CD166 and CD44, among 148 integral membrane or membrane-anchored proteins and 159 membrane-associated proteins. Twenty-nine integrins and cell adhesion molecules, 20 receptors and 18 Ras-related small GTPases were also identified. Upon osteoblast differentiation, the expression levels of 83 proteins increased by at least two-fold, whereas the levels of another 21 proteins decreased by at least two-fold.

DIGE-LC coupled with tandem MS analysis of the plasma membrane-containing fraction from bone marrow MSCs differentiated towards adipocytes allowed Jeong *et al*. [30] to identify 707 proteins, approximately half of which could be identified as membrane-related proteins. Of particular interest was a subset of ectodomain-containing membrane-bound proteins, which encompasses most known surface markers for MSCs, but also contains a multitude of solute carriers and ATPases. Upon adipogenic differentiation, this proteomic profile was amended to include several proteins involved in lipid metabolism and trafficking, at the expense of, most noticeably, ectoenzymes.

Zhang *et al*. [31] analyzed protein expression profiles of undifferentiated, as well as osteogenic-induced MSCs, using 2DE-MALDI-TOF-MS to investigate the early gene expression in osteoblast differentiation. They generated proteome maps of undifferentiated human MSCs and osteogenic-induced human MSCs on day 3 and day 7. One-hundred two spots with at least two-fold changes in expression and 52 differently expressed proteins were successfully identified. These proteins were classified into more than seven functional categories: metabolism, signal transduction, transcription, calcium-binding protein, protein degradation, protein folding, and others.

Kim *et al*. [32] focused on proteins that were differentially expressed during osteogenic differentiation of MSCs from umbilical cord blood. They analyzed the protein expression inherent to osteogenic differentiation with 2DE-ESI-Q-TOF. Eleven differentially expressed spots were observed between the two groups, before and after differentiation; four proteins were found to be involved in the osteogenic process for the first time: PGAM1, VBP1, hsp27 and β-actin. β-actin might also prove useful as a cytosolic biomarker protein for osteogenesis and could be employed in the quality control of osteoblasts for cell-therapy applications.

Proteomic analysis of human MSCs derived from adipose tissue undergoing osteoblast differentiation was realized by Giusta *et al*. [33]. Phenotypic modifications were observed during the *in vitro* osteogenic differentiation process using DIGE-MALDI-TOF/TOF-MS/MS towards osteoblast-like cells. A total of 51 differentially expressed proteins were identified when comparing the three observed conditions; 16 of these proteins were identified, five of which were overexpressed in the early stages of osteogenic differentiation. All five, superoxide dismutase, lamin A, filamin, heat shock protein-27, cathepsin D and fibulin 1, play a very important role in the formation of osteoprogenitor cells. The identification of these proteins opened new ways for their use as biomarkers for the detection of cells undergoing osteogenesis.

Kim *et al*. [34] realized 2DE-MALDI-TOF-MS to study the direct differentiation of MSC from umbilical cord blood towards osteoblasts. They found the 308 spots that were identified during the differentiation process. Sixteen of these proteins were identified with a mean OD (optical density) ratio >30 and were acting in the extracellular region, cytosol or mitochondria, while 20 of these proteins with a mean OD ratio <0.1 had high catalytic activity. These results provide an initial proteomic database for umbilical cord blood MSCs differentiation.

Human MSCs differentiated towards chondrocytic cells with conditioned medium derived from porcine notochordal cells in native tissue or in alginate beads, and compared with chondrogenic (TGFβ-3) or basal medium, were studied by Purmessur *et al*. [35]. Dried peptides subjected to LC-coupled MS/MS for detection indicated the highest levels of glycosaminoglycan (GAG), as well as the upregulation of SOX9 and Collagen II gene expression in MSCs differentiated towards chondrocyte cells in medium from porcine notochordal cells.

Protein phosphorylation plays a critical role in the signaling transduction network during early human MSCs osteogenic lineage commitment. Human MSCs cultured in osteogenic induction medium were analyzed using LC-coupled MS/MS by Lo *et al*. [36]. They observed a dramatic loss of the protein phosphorylation level after one day of osteogenic induction. Pathway analysis of the resulting phosphoproteins revealed a high correlation with cell proliferation and protein synthesis pathways. During osteogenic differentiation, differentially expressed phosphoproteins demonstrated the dynamic alterations in cytoskeleton at early stages of differentiation.

De la Fuente *et al*. [37] followed protein profile changes during the chondrogenic differentiation process of MSCs from umbilical cord stroma using DIGE-MALDI-TOF/TOF-MS/MS. A total of 97 spots were modulated during the chondrogenesis process; 54 of these spots were identified as 39 different proteins and 15 isoforms. Of the 39 different proteins identified, 15 were downregulated, 21 were upregulated and three were up- and down-regulated at different phases of the chondrogenic process.

Rocha *et al*. [38] applied the SILAC technique for the quantitative analysis of protein modulation during the chondrogenic differentiation process of human MSCs from bone marrow. They could identify 622 different proteins by LC-MALDI-TOF/TOF-MS/MS analysis and found 65 proteins whose abundance was significantly modulated between day 2 and day 14 of chondrogenesis. Fibronectin, gelsolin, vimentin, alpha-ATPase, mitochondrial superoxide dismutase and cyclophilin A were increased at day 14 compared to day 2 of chondrogenic induction, thus being markers of the enhanced extracellular matrix synthesis, cell adhesion, metabolism and response to stress processes that take place in the early steps of chondrogenesis.

Herencia *et al*. [39] evaluated the role of Wnt/β-catenin activation during human MSC differentiation into hepatocytes. The differentiation to hepatocytes was achieved using two different conditioned media. Comparison of both differentiation protocols by DIGE revealed the differential expression of 11 proteins with altered expression in hepatocellular carcinoma. In one of these protocols, β-catenin nuclear translocation and the upregulation of genes related to the Wnt/β-catenin pathway, such as Lrp5 and Fzd3, as well as the oncogenes, c-myc and p53, were observed. In the other protocol, Wnt/β-catenin was inactivated. Hepatocytes with nuclear translocation of β-catenin also had abnormal cellular proliferation and expressed membrane proteins involved in hepatocellular carcinoma, metastatic behavior and cancer stem cells. Further, these cells had also an increased auto-renewal capability, as shown in a spheroid formation assay. Cathepsin B and D, adenine phosphoribosyltransferase, triosephosphate isomerase, inorganic pyrophosphatase, peptidyl-prolyl *cis*-trans isomerase A or lactate dehydrogenase β-chain were upregulated only with the protocol associated with Wnt signaling activation, while other proteins involved in tumor suppression, such as transgelin or tropomyosin β-chain, were downregulated in this protocol.

Alves *et al*. [40] used SILAC to study the effect of activin A on the osteogenic differentiation of MSCs from bone marrow. They found 104 proteins changed more than 1.5-fold following activin A treatment. More than half of these proteins, 74 proteins, were downregulated by activin A, while only 30 proteins were upregulated. They observed changes in the expression of collagen XII, osteonectin and several cytoskeleton-binding proteins. Moreover, in osteoblasts differentiated from MSCs, matrix vesicle production was deficient, containing a very low expression of annexin proteins.

Proteomics provides, therefore, very valuable information in directed differentiation studies of MSCs. All the works above have generated new knowledge on the metabolic pathways modulated during the differentiation, thus permitting a better understanding of the processes. The precision and accuracy of the techniques allow the detection of very subtle changes in the expression of proteins or in their phosphorylation state. It is also remarkable that proteomics has permitted the detection in cultured MSCs of the activation of oncogenic factors by certain culture conditions, an undesirable process when it comes to the use of these cells in therapeutic treatment.

## 6. Cellular Therapy with Mesenchymal Stem Cell

MSCs have emerged as a promising tool for treating degenerative or incurable diseases. Proteomic techniques provide a comprehensive basis for understanding the potential effect of MSCs on tissue repair and regeneration. Knowledge of the molecular mechanisms governing the different adult lineages differentiation process is critical to the development of therapeutic applications for human diseases.

Seshi B *et al*. [41] reported a 2D-DIGE protocol in which complex protein samples from normal and leukemic human bone marrow mesenchymal progenitor cells were used as model samples for a combination of liquid-phase IEF with DIGE. Using liquid-phase IEF, the normal and leukemic cells were pre-fractionated into five sub-proteomes after multiplexing, but prior to DIGE. This analysis mapped protein identities to 128 mesenchymal progenitor cell proteins with at least one unique peptide match at >95% confidence. Of these proteins, 72 (56%) were expressed more than 1.25-fold higher or lower in leukemic cells compared with normal cells (*p* < 0.05). These data were used to infer gene ontology biological processes that may be altered in leukemic bone marrow mesenchymal progenitor cells.

The first proteomic analysis of human MSCs after exposure to shear stress was realized by Yi *et al*. [42] using 2DE and MALDI-TOF-MS. Overall, 32 protein spots were identified with high confidence. Thirteen of these proteins were found to be consistently regulated by over two-fold after 3 dyn/cm^2^ shear stress treatment for six hours; 10 were upregulated and three downregulated.

DIGE-MALDI-TOF/TOF was utilized by Zhuang *et al*. [43] to analyze the differential proteome of bone marrow-derived MSCs from adolescent idiopathic scoliosis. A total of 41 significantly altered protein spots were detected, of which 34 were identified, representing 25 distinct gene products. Among these proteins, five related to bone growth and development, including pyruvate kinase M2, annexin A2, heat shock 27 kDa protein, γ-actin, and β-actin, were found to be dysregulated. At the protein level, the results supported the previous hypothesis that the decreased osteogenic differentiation ability of MSCs is one of the mechanisms leading to osteopenia in adolescent idiopathic scoliosis.

Recent studies have shown that microvesicles from MSCs contribute to the recovery of damaged tissues in animal disease models. Kim *et al*. [44] profiled the MSC microvesicles proteome from bone marrow to investigate its therapeutic effects. Seven-hundred thirty proteins were identified by LC coupled MS/MS analysis of MSC microvesicles separated by SDS-PAGE. This proteome included five positive and two variable known markers of MSCs, but no negative markers. In addition, 43 surface receptors and signaling molecules controlling self-renewal and differentiation of MSCs were identified. This analysis showed that cellular processes represented by the MSC microvesicle proteins include cell proliferation, adhesion, migration and morphogenesis. The integration of the self-renewal of MSCs and differentiation-related genes that can be associated with the therapeutic effects of MSC microvesicles includes: surface receptors; signaling molecules (CDC42 and VAV2; cell adhesion); and MSC-associated antigens (CD109, CD151, CD248 and CD276). These proteomes provide a comprehensive basis for understanding their potential effect on MSC microvesicle tissue repair and regeneration.

Dynamic changes in the phosphoproteomic profiles of human MSCs during osteogenic differentiation and revealed potential candidates mediating the osteogenic commitment of human MSCs shown by liquid chromatography tandem mass spectrometry [36] may shed light on the development of new therapeutic targets for metabolic bone diseases, such as osteoporosis and osteomalacia.

Han S *et al*. [45] studied differential proteins expressed in the MSCs of patients with degenerative scoliosis. They compared MSCs from patients with degenerative scoliosis and patients with lumbar spinal stenosis. The MSC samples were analyzed by DIGE-MALDI-TOF-MS to find the differential proteins. They found 115 spots that were expressed differently in the MSCs of degenerative scoliosis patient; 44 proteins were identified. Of these proteins, PIAS2, NDUFA2 and TRIM 68 were upregulated in degenerative scoliosis. This information from this proteomics analysis will be useful in understanding the pathophysiology of degenerative scoliosis and opens further lines of investigation on the functional pathway, the specificity and the mechanism of action of these proteins.

Proteomic profiling has provided a variety of novel molecular procedures that can form the basis for more in-depth investigations into the effects of shear stress *in vitro* human MSCs proliferation, differentiation and apoptosis; this may, in turn, significantly influence applications in stem cell therapy and tissue regeneration.

## 7. Outlook and Perspectives

Liquid chromatography-multiple reaction monitoring mass spectrometry of peptides using stable isotope dilution [53] provides a powerful tool for targeted protein quantification. However, the high cost of labeled peptide standards for stable isotope dilution (SID) is an obstacle to multiple reaction monitoring studies. Zhang *et al*. [53] compared SID to a labeled reference peptide (LRP) method, which uses a single labeled peptide as a reference standard for all measured peptides, and a label-free (LF) approach, in which quantification is based on the analysis of un-normalized peak areas for detected MRM transitions. They concluded that the LRP and LF methods provide cost-effective alternatives to SID for many quantitative liquid chromatography-multiple reaction monitoring mass spectrometry applications. These procedures have not yet been applied to study the application of MSCs to cell therapy, but offer this opportunity. Proteomic differential displays could help to increase the cell therapies with MSCs through specific adjustments based on these displays.

Promising techniques need special mention here. Matrix assisted laser desorption ionization mass spectrometry imaging (MALDI-MSI) and laser micro-dissection are currently under expansion and development. The MALDI-MSI combines high resolution power and the ability to monitor a large number of proteins in a single analysis feature of the mass spectrometry and has the advantage of being able to apply histomorphologic techniques, including providing visual information about the spatial distribution of analytes. The approach is as simple as a laser “sweep” with a given spatial resolution, 50 microns or less, of a tissue, cut with histological techniques for mass spectrometry, to determine the intensity at each pixel of a certain range of *m/z*. In practice, this involves making a two-dimensional map of the distribution of ions (peptides, lipids, sugars) in a histological section. Although, at first, it was successful only in soft tissues (brain, kidney, lung, *etc*.) [54,55], investigators have recently begun to utilize it successfully in tissues, such as cartilage [56]. The laser capture micro-dissection (LCM) technique identifies LCM regions corresponding to individual cells or groups of cells in tissue sections, using a laser coupled to a microscope. The resulting sections are deposited in micro-tubes for analysis, in this case, an extraction of proteins that can be further examined with differential labeling to, for example, determine the differential proteome of cells or groups of adjacent cells in the same tissue [57]. This technique opens possibilities to study small groups of MSCs undergoing cell division after differentiation.

## 8. Conclusions

To summarize, applied mass spectrometry proteomics has enabled a breakthrough in resolving the power and speed of analysis. Few techniques have proven valid for detection, analysis and quantification, in a single experiment, of many analytes; mass spectrometry is one of them. Thus far, 2D-based proteomic strategies have been primarily used to characterize MSCs. In the future, it will be essential to use gel-free-based strategies to delve deeper into the characteristic proteomes of different MSC populations. While increasingly specific and sensitive equipment is launched on the market every year, clinical validation by other, even more sensitive techniques, such as RT-PCR and immunoassays, is still needed.

## Abbreviations

DIGE: difference in-gel electrophoresis
2D-PAGE: two-dimensional polyacrylamide gel electrophoresis
ESI: electrospray ionization
GELFREE: gel-eluted liquid fraction entrapment electrophoresis
IEF: iso-electric focusing
IMAC: immobilized metal ion affinity chromatography
iTRAQ: isobaric tags for relative and absolute quantification
LC: liquid chromatography
LCM: laser capture micro-dissection
MALDI-MSI: matrix-assisted laser desorption ionization mass spectrometry imaging
MSCs: mesenchymal stem cells
^18^O: (18)O-labeling of reactive carbonyl modifications
PCR: polymerase chain reaction
PMF: peptide mass fingerprinting
SELDI-TOF-MS: surface enhanced laser desorption/ionization-time of flight-mass spectrometry
SCX: strong cation exchange
SDS-PAGE: sodium dodecyl sulfate polyacrylamide gel electrophoresis
SID: stable isotope dilution
SILAC: stable isotope labeling by/with amino acids in cell culture
Ti^20^: tolerable intake 20
